# Successful treatment of nonunion in severe finger injury with low-intensity pulsed ultrasound (LIPUS): a case report

**DOI:** 10.1186/1752-1947-6-209

**Published:** 2012-07-18

**Authors:** Michaela Huber, Lukas Prantl, Sebastian Gehmert

**Affiliations:** 1 Department of Trauma, Plastic & Hand Surgery, University Medical Center Regensburg, Regensburg, Germany

## Abstract

**Introduction:**

Severe injuries of the hand or single fingers require immediate treatment but surgical fixation methods are limited depending on soft tissue damage. Thus, it is very common that severe soft tissue damage along with poor osteosynthetic bone fixation results in a delayed healing process or nonunion. Low-intensity pulsed ultrasound (LIPUS) has been proven to stimulate bone formation in *in vitro* studies and also to significantly accelerate nonunion healing in animal studies and clinical trials but to date there are no data with respect to nonunion in phalanx fracture.

**Case presentation:**

We report a case in which we successfully used LIPUS in a 19-year-old Caucasian man with a nonunion of his ring finger after injury and first treatment with K-wire osteosynthesis.

**Conclusion:**

We recommend that LIPUS be considered as an option to treat nonunions in fractures of the hand, especially because it is a soft tissue conserving method with a good functional result.

## Introduction

In recent years, low intensity pulsed ultrasound (LIPUS) has been the focus of several studies examining how to help stimulate bone formation in fractures and nonunions [1-3]. However, to the best of our knowledge, there have been no reported studies dealing with the use of LIPUS to treat fractures or nonunion of the fingers.

Up to now in cases of nonunions, trauma surgeons have had to perform additional surgery with a more stable osteosynthesis and bone grafting. This can cause additional complications to the already pre-damaged soft tissue with further loss of function [4,5]. In this case we successfully used LIPUS, a non invasive treatment, to investigate its applicability in treating nonunion of fingers.

## Case presentation

**Figure 1:**
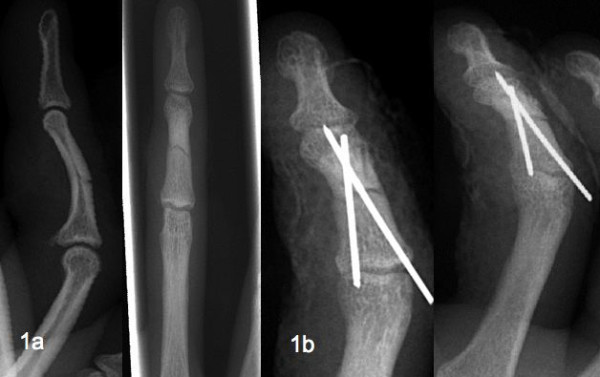
Initial X-ray after injury (a) and control X-ray after the osteosynthesis (b).

A 19-year-old Caucasian man was admitted to our emergency department with a critical ischemia of his right ring finger due to a crushing injury. His finger had been caught between a car and a car-jack while he was working as a mechanic. The first physical examination of the ring finger revealed an open wound of 3 cm on the dorsal side of the middle phalanx. Edges of the wound reached the ulnar and radial digital neurovascular bundle. The patient reported decreased sensibility of the end phalanx including painful range of motion (ROM) but without loss of function of all tendons. We found a restricted finger blood flow detected by nail bed compression in comparison with the uninjured fingers. An X-ray showed an undislocated transverse fracture of the middle phalanx (Figure 1a). The patient was immediately transferred to the operating room (OR) where the wound was examined. Severe damage of the surrounding soft tissue was seen. Microscopic examination revealed no defect of the crushed vessels, the radial digital nerve was intact, but an epineural lesion was apparent for the ulnar digital nerve. A minimal osteosynthesis with two Kirschner-wires (K-wires) was performed. X-ray imaging showed an anatomical retention and fixation of the fracture (Figure 1b). The dorsal wound of the ring finger was cut out and sutured. The patient was discharged from the hospital on the fourth day without any sign of wound healing disturbance. The ring finger was immobilized with a finger splint including the proximal interphalangeal joint (PIP) and distal interphalangeal joint (DIP).

The patient was seen five months after initial treatment in our Out-Patient Clinic and reported painful moving of the right ring finger. The examination showed an instability of the middle phalanx with a passive lateral movement. Twenty degrees ulnar deviation of the ring finger with decreased ROM of the PIP joint (E/F 0-0-60˚) was apparent. In addition, the ring finger tended to cross over and overlap the adjacent finger when making a fist. The patient reported that ulna deviation of the ring finger started when K-wires had been removed by an office-based orthopedic surgeon two months after the initial surgery.

**Figure 2:**
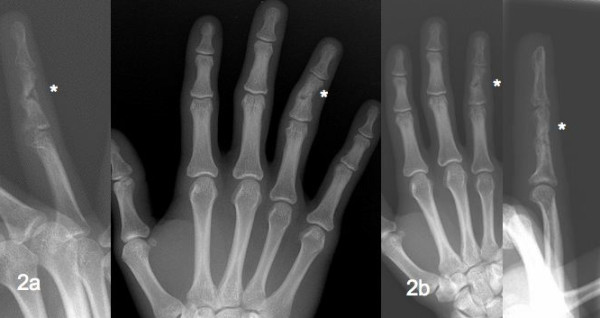
Control X-ray five months after initial operation (a); X-ray six weeks after LIPUS therapy (b).

Since that time the finger was fixed again with a splint. He also complained about tingling, coolness and numbness of the injured finger. Since the X-ray showed a nonunion (Figure 2a) we started treatment with a LIPUS device (Fa. Melmak GmbH, Munich, Germany) three times daily for 20 minutes including the following parameter configuration:

Average intensity I = 30 mW/cm² (SATA), ultrasound frequency F = 1.5 MHz, signal impulse duration 200 microseconds, repetition rate 1 kHz, effective radiating area 3.88 cm^2^, temporal average power 117 mW.

**Figure 3:**
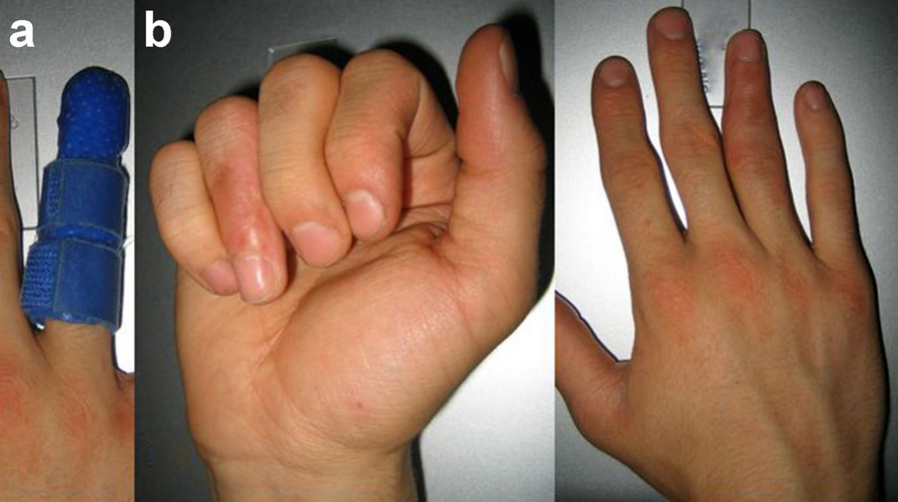
Personalized thermoplastic splint (a) and the functional result eight months after the initial trauma (b).

The ring finger was immobilized with a fitted personalized thermoplastic splint (Figure 3a) for six weeks. Six weeks after LIPUS treatment the patient reported to be without pain when moving his ring finger and clinical examination revealed a slight persistent ulna deviation of five degrees. The clinical examination showed a stable middle phalanx compared to the result six weeks previously. After four weeks of physiotherapy the ROM of the PIP joint was E/F 0-0-85° and the fist closure was full (Figure 3b), except for the pre-existent limited ROM of the DIP-Joint (E/F 0-0-15˚). An X-ray confirmed fracture healing and calcification of the soft callus was clearly evident (Figure 2b).

## Discussion

Severe injury of the fingers or the hand with a phalanx fracture requires rigid fixation with anatomical reduction to achieve appropriate bone healing. In addition, stable fracture fixation facilitates exercise very soon after surgical treatment avoiding restricted movement. The initial soft tissue damage, however, can limit the approach of osteosynthesis [4-6]. Using K-wires reduces the risk of further damage to the surrounding tissue while accepting less compression of the fracture that might cause nonunion. Second surgery is very common in nonunion fracture healing including bone graft and plate fixation [7] but second surgical intervention causes new or repeated soft tissue damage, leading to further loss of function. However, phalanx fracture fixation is needed in most cases and should be limited to a short period of time in order to achieve a ROM without restriction. Thus, low-intensity ultrasound seems to be an additional favorable tool for nonunion since it is effective and non-invasive [2,3]. Gebauer *et al*. reported in a randomized prospective trial that daily therapy of 20 minutes with low-intensity ultrasound achieved bone healing in 85 % of cases [8] which was confirmed by other studies [9-12]. In a recently published Cochrane Review about ultrasound and shockwave therapy, in which 12 studies were evaluated, the authors concluded that the evidence of the analyzed clinical trials is insufficient to support the routine use of LIPUS [1]. In this case, there had been a three month splinting without success, so we decided to use LIPUS instead of bone grafting and osteosynthesis.

LIPUS is a pain free therapy performed daily at home by the patient with the possibility of avoiding a second surgical procedure. This out-patient treatment reduces the length of hospital stays for patients and the expenses of the health care system [13].

## Conclusion

We report successful ultrasound treatment after inadequate bone healing with insufficient stability of the middle phalanx. The patient suffered from reduced ROM in the DIP and PIP joints. The patient also had an ulnar deviation associated with pain owing to the instability of the fracture. Based on this case report we suggest LIPUS as a possible approach for nonunion in finger injuries, however further clinical trials are necessary to confirm these preliminary findings.

## Consent

Written informed consent was obtained from the patient for publication of this case report and any accompanying images. The written consent is available for review by the Editor-in-Chief of this journal.

## Competing interests

The authors declare that they have no competing interests.

## Authors’ contributions

MH was the main author and performed the clinical assessment, follow-up and the bibliographic research. LP performed the clinical assessment, the surgery and the follow-up. SG was a major contributor in writing the manuscript. All authors have read and approved the final manuscript.
